# Resonantly Enhanced Emission from a Luminescent Nanostructured Waveguide

**DOI:** 10.1038/srep34396

**Published:** 2016-09-29

**Authors:** Yasuhisa Inada, Akira Hashiya, Mitsuru Nitta, Shogo Tomita, Akira Tsujimoto, Masa-aki Suzuki, Takeyuki Yamaki, Taku Hirasawa

**Affiliations:** 1 Advanced Research Division, Panasonic Corporation, 1006 Kadoma, Kadoma City, Osaka 571-8501, Japan

## Abstract

Controlling the characteristics of photon emission represents a significant challenge for both fundamental science and device technologies. Research on microcavities, photonic crystals, and plasmonic nanocavities has focused on controlling spontaneous emission by way of designing a resonant structure around the emitter to modify the local density of photonic states. In this work, we demonstrate resonantly enhanced emission using luminescent nanostructured waveguide resonance (LUNAR). Our concept is based on coupling between emitters in the luminescent waveguide and a resonant waveguide mode that interacts with a periodic nanostructure and hence outcouples via diffraction. We show that the enhancement of resonance emission can be controlled by tuning the design parameters. We also demonstrate that the enhanced emission is attributable to the accelerated spontaneous emission rate that increases the probability of photon emission in the resonant mode, accompanied by enhanced the local density of photonic states. This study demonstrates that nanostructured luminescent materials can be designed to exhibit functional and enhanced emission. We anticipate that our concept will be used to improve the performance of a variety of photonic and optical applications ranging from bio/chemical sensors to lighting, displays and projectors.

Techniques for controlling photon emissions have attracted much research attention due to their potential for application ranging from photonic devices to more commonly used optical devices^1^. Many studies have demonstrated that spontaneous emissions can be modified by varying the local density of photonic states (LDOS). Microcavities^2^,^3^, photonic crystals^4^,^5^ and plasmonic structures such as plasmonic nanocavities^6^,^7^ and nanoantennas^8^,^9^ have been explored to modify spontaneous emission by confining photons within a space smaller than their optical wavelength. These techniques enable high controllability of photon emission through a precise overlap between the emitter and the spatially-confined resonant mode, and therefore have major potential for use in photonic devices such as low-threshold lasers and single-photon sources. In contrast, metallic nanoparticles^10^,^11^ and nano-metallic arrays^12^,^13^ have been investigated with the aim of achieving enhanced emissions with high brightness by utilizing multiple emitters; however, the inherent losses due to plasmon resonance limit their performance. In this work, we propose the use of nanostructured waveguide resonance (LUNAR) to enable the simultaneous control of a number of emitters with high efficiency.

## Results

### Characterization of nanostructured waveguide resonance

**Figure 1:**
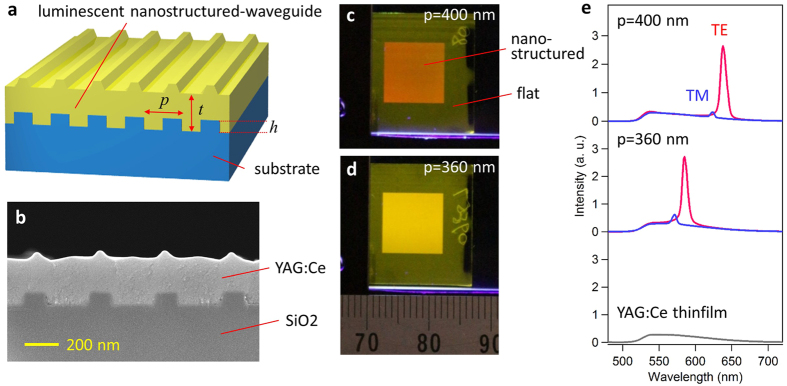
LUNAR structure. (**a**) Schematic illustration of a LUNAR structure. (**b**) Cross-sectional SEM image of a LUNAR sample. The sample was fabricated by depositing YAG:Ce phosphor with *t* = 250 nm on the SiO_2_ nanograting substrate with *p* = 400 nm and *h* = 80 nm. (**c,d**) Pictures of LUNAR samples with *p* = 400 and 360 nm, excited by a 450-nm LED under ambient room conditions. A ruler with units of mm is shown as a reference scale. **(e**) Measured photoluminescence spectrum of LUNAR structure for TE and TM polarizations, together with the spectrum of YAG:Ce thin film for comparison.

Our design concept is based on coupling between emitters and guided mode resonance^14^,^15^, which is supported by the luminescent nanostructured material and thus can be controlled by structural parameters. Figure 1a depicts our basic design of a LUNAR structure. The luminescent nanostructured-waveguide is supported by the substrate. The LUNAR samples can be characterized by three design parameters: Period *p*, height *h* of the nanograting, and thickness *t* of the luminescent waveguide. Our samples were fabricated by depositing YAG:Ce phosphor, which is widely used in white LEDs for displays and lighting, onto a 1D nanograting fabricated on top of a silica substrate (see Methods). The resonant waveguide modes are supported by the nanostructured phosphor film due to the refractive index of YAG:Ce (~1.77) being higher than that of silica (~1.45). Figure 1b shows a scanning electron microscope (SEM) image of the sample. Figure 1c,d are photographs of the samples excited by a blue LED. Resonant emissions are observed in the photoluminescence spectra, and the resonant wavelength varies according to period and polarization (Fig. 1e).

**Figure 2:**
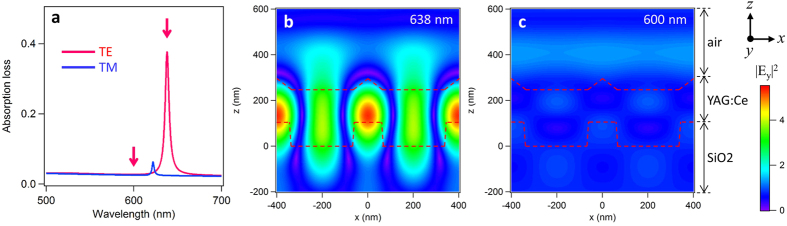
Calculation of resonance mode. (**a**) Calculated resonance spectrum of a LUNAR structure with (*p, h, t*) = (400 nm, 80 nm, 250 nm) for normal incidence (-z direction) using RCWA. The model structure is constructed based on the SEM image shown in Fig. 1b (see Supplementary Information Fig. S2). (**b,c**) The electric field amplitude |*E*_y_|^2^ for TE mode resonance and off-resonance whose conditions correspond to the arrows in (**a**). The red dashed lines indicate the boundaries of the material models.

To characterize the resonant modes in the LUNAR structure, we calculated the resonance spectrum and electric field distribution using rigorous coupled wave analysis (RCWA). In an optical resonance system, resonant modes appear in the absorption and scattering spectra^12^,^16^,^17^. In our calculation, we took this optical loss into account by setting the extinction coefficient for the model material instead of for the scattering centers in the YAG:Ce of the LUNAR sample. The detailed descriptions are shown in Supplementary Fig. S4. Figure 2a shows the calculated resonance spectrum of the LUNAR structure. The resonant peaks for the TE and TM polarizations are similar to the peaks seen in the emission spectrum shown in Fig. 1e. Figure 2b,c show the distribution of electric field amplitude calculated at the resonance (638 nm), together with that of an off-resonance wavelength (600 nm) for comparison. The electric field is strongly enhanced at the resonance: its distribution is similar to a standing wave, with the nodes located precisely along the periodic nanostructure. This suggests that the optical field is confined as a quasi-waveguide mode which is formed from the waveguide light that interacts with the nanostructure.

**Figure 3:**
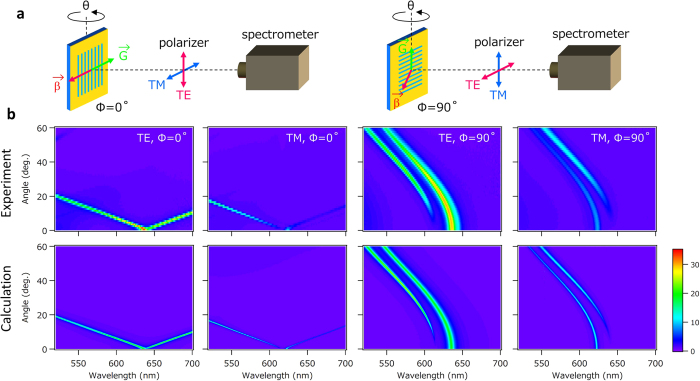
Angle-dependence of LUNAR emission. (**a**) Schematics showing the experimental configuration of the sample orientation for detection of polarization. *β* denotes the propagation vector of the waveguide mode and *G* the grating vector of the periodic nanostructure. (**b**) Angle-dependence of LUNAR emission enhancements for different polarizations and rotation configurations. The measured (calculated) enhancements of the LUNAR emission are obtained from the emission (loss) spectrum for the sample with (*p, h, t*) = (400 nm, 100 nm, 250 nm) normalized by that of the thin film with a flat surface. The scale bar is for all figures.

The quasi-waveguide mode is strongly diffracted by the periodic nanostructure because it propagates with its phase matched to the period of the nanostructure. This diffraction is the origin of the LUNAR emission. The wavelength and the output direction of the LUNAR emission are determined by the configuration of the direction of the propagation vector of the waveguide light and the grating vector of the periodic nanostructure. To clarify this relationship, we measured the angle dependence of the wavelength of the LUNAR mode. Figure 3a illustrates the experimental configurations of the measurements, and Fig. 3b shows the measured enhancement of the emission and the calculated enhancement of loss for the incidence. When we consider the angle dependence along the axis perpendicular to the grating vector (*Φ* = 0), the diffraction can be simplified as a grating equation in the waveguide plane: *k*_||_ = *β* ± *mG*, where *k*_||_ (=*k*_0_sin*θ, k*_0_ is the wavenumber in free space and *θ* is the output angle from surface normal) denotes the in-plane wave vector component, *β* (=*k*_0_*n*_eff_, *n*_eff_ being the effective refractive index) the propagation constant, *m* the order of diffraction, and *G* (=2π/*p, p* being the period of the nanostructure) the grating vector. For the first-order diffraction (*m* = 1), this equation is simplified to *λ*_0_ = ±*p*(*n*_eff_−sin*θ*) where *λ*_0_ (=2π/*k*_0_) is the free space wavelength. We can confirm that the resonant modes observed in Fig. 3b exhibit similar electric field distributions to those in Fig. 2b, independently of the resonant wavelength. This indicates that *n*_eff_ is almost entirely independent of the resonant wavelength when *n*_wav_ has a small dispersion. Under this assumption, the resonant wavelength should show a linear dispersion with d*λ*/d*θ *~ 7 nm/deg. for small values of *θ*. This agrees with the results in Fig. 3b. An examination of angle dependence along the axis parallel to the grating vector (*Φ* = 90) shows that the emissions which result from the diffraction in the waveguide mode with the in-plane wave vector *β* are not parallel to the grating vector *G*. This causes parabolic dispersion. As concerns polarization dependence, the TE mode has a longer resonant wavelength, since the effective optical path length in the TE mode is longer due to the larger phase shift caused by total internal reflection^18^.

### Characteristics of LUNAR enhancement

The field enhancement induced by the photon confinement effect due to the quasi-waveguide mode directly corresponds to the enhancement of LDOS. Due to this large LDOS, emitters in the LUNAR structure experience modification of spontaneous emission. According to Fermi’s golden rule, the spontaneous emission rate *γ*_emi_ will depend on the LDOS; and, in an optical resonant system, *γ*_emi_ is proportional to *Q*/*V*_mode_ where *Q* is the quality factor (Q-factor) and *V*_mode_ is the mode volume^19^,^20^,^21^. In the LUNAR structure, two decay channels dominate the Q-factor: the diffraction induced by the nanostructure, and the loss caused by the scattering or absorption within the luminescent material. We define these decay rates as the outcoupling rate *κ*_out_ and the loss rate *κ*_loss_, respectively. The Q-factor is given by *Q* = *ω*_0_/(*κ*_out_ + *κ*_loss_) where *ω*_0_ is the resonant frequency.

We investigated the optical confinement effect through measurements of the Q-factor, LDOS enhancement and their relationship with emission enhancement. In a systematic analysis, we prepared a series of LUNAR samples with different height to investigate the *κ*_out_ dependence. All the samples were prepared from the same batch to minimize any fluctuation in YAG:Ce thickness and composition; our results showed the resonant state (wavelength *λ*_0_, mode volume *V*_mode_ and loss rate *κ*_loss_) to be close to identical in all the samples. We conducted two different experiments to evaluate the Q-factor, LDOS enhancement and emission enhancement (see Methods). First, we observed the resonance spectrum through light scattering measurements to evaluate the LDOS. In linear optics, the local electromagnetic field amplitude of a mode is linearly connected to that of a plane wave at a certain point in the far field; therefore, the strength of the local field induced by incident light is directly proportional to the LDOS^22^. Consequently, the LDOS can be evaluated as a function of enhancement of the loss (scattering or absorption) for the incidence because the loss is a function of the field amplitude. We also performed photoluminescence (PL) experiments to qualify the emission enhancement. The resonant emission is likely to be dominated by the increased spontaneous emission rate to the resonant mode accompanied by a large LDOS. The probability of photon emission to this mode will thus be enhanced in combination with decreased emission probability in normal modes due to energy conservation in the excited emitter.

**Figure 4:**
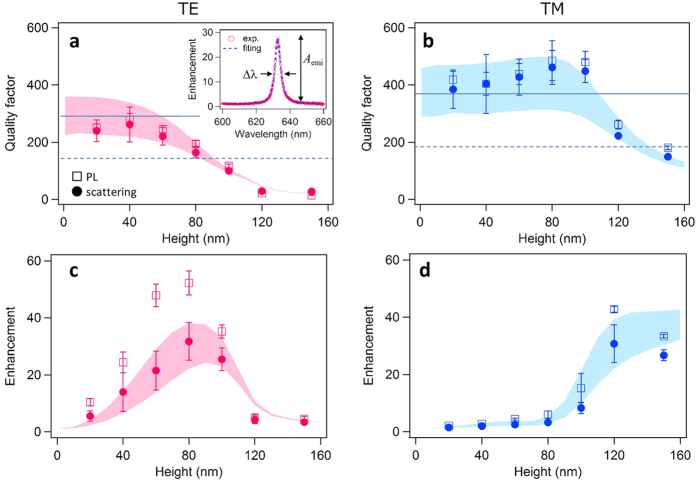
LUNAR enhancement characteristics. (**a,b**) Quality factors for TE and TM modes versus height *h* of a LUNAR structure with (*p, t*) = (400 nm, 250 nm). Symbols and error bars represent means and standard deviations of the measured values obtained from the scattering and PL experiments. The shaded areas show the predictions of the calculation with the waveguide loss rate *κ*_loss_ that is set by the extinction coefficient of the luminescent material ranging from 0.0025 to 0.004. The inset shows the typical enhancement spectra obtained from the PL spectra of the LUNAR sample normalized by that of YAG:Ce film with the fitted Lorentzian curve to extract the emission enhancement *A*_emi_ and the line width Δ*λ* for *Q = λ*_0_/Δ*λ*. The solid lines indicate *Q*_0_ (=*ω*_0_/*κ*_loss_), which is solely determined by the loss rate *κ*_loss_, and the broken lines indicate *Q*_0_/2, which corresponds to the critical coupling regime where *κ*_loss_ is in balance with the coupling rate *κ*_out_. (**c,d**) Enhancement versus height *h*. The enhancement *A*_loss_ of scattering loss corresponds to the LDOS enhancement, while the PL experiment measures the emission enhancement *A*_emi_. Both enhancement curves show their maximum value near the critical coupling. The deviation of the PL enhancement *A*_emi_ from the scattering enhancement *A*_loss_ and the calculation suggests that the probability of photon emission in the resonance mode is enhanced due to the accelerated emission rate.

Figure 4a–d show the Q-factor and the enhancement of LDOS and emission versus the height *h* of the LUNAR structure. The inset shows typical enhancement spectra of LUNAR emission with the fitted curve to extract the Q-factor *Q* (=*λ*_0_/*Δλ*) and the emission enhancement *A*_emi_. We also obtained *Q* and the scattering enhancement *A*_loss_ in the same manner for the scattering spectrum. The symbols indicate the data obtained from the scattering and PL experiments and the shaded areas represent the theoretical prediction of *Q* and *A*_loss_ obtained from the calculated resonance spectrum (see Supplementary Information). The measured Q-factors from the scattering and PL experiments show coincidence and also agree with the theoretical predictions (Fig. 4a,b). The Q-factor decreases with larger *h*, which suggests that the outcoupling rate *κ*_out_ increases according to *h*. In Fig. 4c,d, the *A*_loss_ curve in the scattering experiment shows agreement with the calculation, whereas the *A*_emi_ from the PL experiment shows a significantly larger value than the *A*_loss_. These results suggests that, as mentioned above, LUNAR emission (*A*_emi_) can be attributed not only to the modified directional pattern of the emission in accordance with the LDOS (*A*_loss_), but also to the increased spontaneous emission rate, which enhances the probability of photon emissions to the resonant mode.

The LUNAR emissions measured in the PL experiments represent only part of the waveguide light from the emitter. We defined the extraction efficiency as *K*_emi_ = *κ*_out_/(*κ*_out_ + *κ*_loss_), which corresponds to the ratio of outcoupled light to the emitted light. The value for emission enhancement *A*_emi_ is obtained from the product of the emission rate *γ*_emi_∝*Q*/*V*_mode_ and the extraction efficiency *K*_emi_, which gives *A*_emi_∝(*ω*_0_/*V*_mode_)*κ*_out_/(*κ*_out_ + *κ*_loss_)^2^. Assuming constant resonant wavelength and mode volume, it can be simplified to *A*_emi_∝*κ*_out_/(*κ*_out_ + *κ*_loss_)^2^. This *A*_emi_ reaches a maximum when *κ*_out_ = *κ*_loss_, which corresponds to the so-called critical coupling condition in a resonant system^23^,^24^,^25^. At the critical coupling, the enhancement *A*_scatt_ should also show a maximum value for the scattering experiment. When *κ*_out_ = *κ*_loss_, the Q-factor takes the half value: *Q*_c_ = *Q*_0_/2 where *Q*_0_ (=*ω*_0_/*κ*_loss_) is the Q-factor when *κ*_out_ = 0, namely, no diffraction structure exists. Figure 4a,b show the critical coupling conditions to be achieved near *h* = 90, 130 nm for TE and TM modes respectively. The enhancement reaches a maximum near the critical coupling, which agrees with our theoretical predictions. The critical coupling of the TM mode that occurred at larger *h* is a result of the lower diffraction efficiency caused by the small overlap of the evanescent field of the waveguide mode with the periodic nanostructure. We confirmed that the PL lifetime does not change significantly. This is because the spontaneous emission is enhanced in only a small spectral range (~1 nm), while YAG:Ce emits light with a broad spectrum (~200 nm).

## Discussion

In conclusion, we have demonstrated resonant emission from a luminescent nanostructured waveguide phosphor. The enhancement of the directional and polarized emission can be attributed to the enhanced spontaneous emission rate, combined with the large LDOS of the resonant mode that is supported by the luminescent nanostructured material. Our results based on theoretical analysis indicate that the enhanced emission can be further improved by reducing the scattering centers in the luminescent waveguide. We anticipate that our results will open up new perspectives in the structural design of luminescent materials that will extend emission functionality and consequently lead to significant developments in the design concepts of a wide variety of photonic and optical devices. For example, the LUNAR structure can be used to improve the performance of PL-based bio/chemical sensors since the refractive index of the surrounded material can be monitored by measuring the enhanced PL. In addition, a LUNAR structure made of emitting material with a narrow bandwidth will realize a highly directional and polarized light source, which would reduce the size of optics as well as improving light utilization efficiency, and consequently lead to significant developments in the design concepts of a wide variety of optical devices. We also anticipate that the LUNAR system can be applied to lasers, nonlinear optics and cavity QED experiments.

## Methods

### Fabrication of LUNAR samples

Our nanograting substrates were prepared using photolithography followed by a dry etching process. A periodic pattern was exposed on photoresist on an 8-inch quartz wafer using an ArF 193-nm exposure tool (ASML1100) with a half-tone phase-shifting mask. The wafers were etched under oxide etching conditions, and the height of the nanograting was controlled by etching time. Next, YAG:Ce phosphor was deposited on the substrate by radio frequency magnetron sputtering. We prepared a 3-inch ceramic target made of YAG:Ce with a composition of (Y_1−x_, Ce_x_)_3_Al_5_O_12_: x = 0.008. The sputtering conditions were optimized to uniformly and symmetrically pack the YAG:Ce into the grating. The sputtering RF power was 200 W. The process gas ratio was Ar/O_2_ = 10:10 at a working pressure of 0.5 Pa. The distance between the target and the substrate carrier was 60 mm. The substrate temperature was held constant at room temperature. After deposition, the samples were annealed at 920 °C for two hours for recrystallization. The annealing atmosphere was N_2_/H_2_ = 99.5:0.5 at a pressure of 1 atm to prevent the oxidation of Ce^3+^. The rising and descending rate of the temperature was set at 200 K/h to minimize heat stress. Our LUNAR samples showed an internal quantum efficiency of ~90%, which was almost entirely independent of the design parameters of the LUNAR structure.

### Light scattering and PL experiments

We observed the scattering and the photoluminescence spectrum in an oblique direction to eliminate the degeneracy that occurred at the normal angle due to the counter-propagating resonant waveguide modes. We chose the resonant mode with a wavelength of 633 nm which appears in the angle within 1–4 degrees in both TE and TM modes (see Supplementary Fig. S4). In the scattering experiment, we measured the scattered light as a function of the angle of incidence of a He-Ne laser (633 nm) by rotating the LUNAR sample along the axis perpendicular to the grating vector. The scattered light was detected by a photodetector from the direction perpendicular to the incidence plane, so as not to pick up the transmitted or reflected beams. The line width Δ*λ* was obtained from the line width Δ*θ* of the scattering spectrum in angle space in consideration of the linear dispersion with d*λ*/d*θ *~ 7 nm/deg. In the PL experiment, we measured the emission spectrum with a solid angle of 0.02°, limited by the aperture. The spectral resolution of this measurement was 0.6 nm, which was determined by the angle-dependent resolution of the LUNAR emission and the resolution of the spectrometer. The data was fitted by a function obtained by a convolution of Lorentzian and Gaussian where the Gaussian part takes care of the experimental broadening with FWHM of 0.6 nm.

## Additional Information

**How to cite this article**: Inada, Y. *et al*. Resonantly Enhanced Emission from a Luminescent Nanostructured Waveguide. *Sci. Rep.* **6**, 34396; doi: 10.1038/srep34396 (2016).

## Supplementary Material

Supplementary Information
