# Supplementary material for: Resonantly Enhanced Emission from a Luminescent Nanostructured Waveguide
Source: Sci Rep. 2016 Sep 29;6:34396. doi: 10.1038/srep34396 (PMC5041154; doi:10.1038/srep34396)
Supplement: Supplementary Information [file srep34396-s1.pdf]

Supplementary Information

Resonantly Enhanced Emission from a Luminescent Nanostructured Waveguide

Yasuhisa Inada[1,*], Akira Hashiya[1], Mitsuru Nitta[1], Shogo Tomita[1], Akira Tsujimoto[1], Masa-aki Suzuki[1], Takeyuki Yamaki[1] and Taku Hirasawa[1]

1 Advanced Research Division, Panasonic Corporation, 1006 Kadoma, Kadoma City, Osaka 571-8501, Japan.
*inada.yasuhisa@jp.panasonic.com

Analysis of LUNAR samples and calculation model

Our LUNAR samples were fabricated by depositing YAG:Ce onto a nanograting substrate. Figure S1 shows cross-sectional scanning electron microscope (SEM) images of LUNAR samples with h = 60, 100, and 150 nm. The surface structures are followed by the nanograting substrates, and show a close to regular periodicity. Both the surface structures and the nanograting with embedded YAG:Ce interact with the waveguide light, and thus affect the performance of the LUNAR emission through the outcoupling rate κ_{out}.

To analyze the resonant modes in the LUNAR structure, we calculated the optical properties using rigorous coupled-wave analysis (RCWA). The calculation models were constructed by approximating the surface structure to a triangular shape, as shown in Figure S2a. The base w_{nano} and the height h_{nano} were read off from the SEM images (Fig. S1). The w_{nano} and h_{nano} versus height of the nanograting (h) are shown in Figure S2b. The dotted lines represent the interpolating curves made by fitting the data with an empirically-chosen sigmoid function.

To set the refractive indices for model materials, we evaluated the refractive indices of the silica substrate and the YAG:Ce thin film using ellipsometry measurements. The index of the silica substrate was 1.45 at 633 nm. For the YAG:Ce, the measured data fitted better when we assumed the index (<1.55) near the silica substrate to be lower than that of the index near the surface (~1.77). This suggests that the material composition near the silica substrate diverges from YAG:Ce. We examined the composition of YAG:Ce film on the substrate using energy-dispersive X-ray spectroscopy (EDX). Figure S3a shows a transmission electron microscope (TEM) image of the sample. Figure S3b shows the distribution of Si impurity at the profile line, marked as the red line in Figure S3a. While the composition near the surface was Y:Al:O = 13:25:61, which is close to that of YAG ($Y_3Al_5O_{12}$), a large amount of Si impurity was detected near the substrate. The composition near the SiO_2 substrate is supposed to be Y-Si-O, Y-Al-O, or mixed crystal of SiO_2 and YAG. This should result in a lower index near the substrate than YAG.

To incorporate this effect in our calculations, we looked at simplified models (Fig. S4a). The YAG:Ce embedded in the nanograting was significantly affected by the Si impurity and its index (n_x) needed to be varied as a function of the nanograting height h. We empirically adopted n_x = 1.55 (a constant) for Model #1, n_x = 1.55 - h/(4 μm) for Model #2 and n_x = 1.55 - h/(2 μm) for Model #3. For air (n_{air}), YAG:Ce near the surface

(n_{YAG}) and silica (n_{sub}), we use the index to make n_{air} = 1, n_{YAG} = 1.77, n_{sub} = 1.45 in all the models. To check the validity of these models, we calculated the emission angle of the LUNAR emissions at a wavelength of 633 nm and compared them with the experimental values. As shown in Fig. S4b, Model #2 closely agreed with the experimental values. We therefore adopted Model #2 for our calculations in Figs. 1 - 4.

Next, we examined the scattering loss of the LUNAR structure. As shown in Fig. S3a, grain boundaries are seen in the YAG:Ce film that cause light scattering in waveguide mode. To investigate this scattering effect, we measured the Q-factor versus YAG:Ce thickness t of the LUNAR samples with p = 400 nm and h = 20 nm (Fig. S5). Since h = 20 nm is sufficiently small, the Q-factor is mainly determined by the scattering loss. The measured Q-factor was almost proportional to the YAG:Ce thickness, which suggests that the scattering loss might be caused not by the surface roughness but by the scattering centers inside the YAG:Ce film. In our calculation model, we accounted for this loss by applying the extinction coefficient of the model material of YAG:Ce, instead of the scattering centers, to simplify the model. To estimate the loss rate, we calculated the Q-factors for different extinction coefficients and compared them with the experimental results in Figure 4. Our calculation, which assumes that the extinction coefficient is between 0.0025 and 0.004, shows excellent agreement with the experimental values.

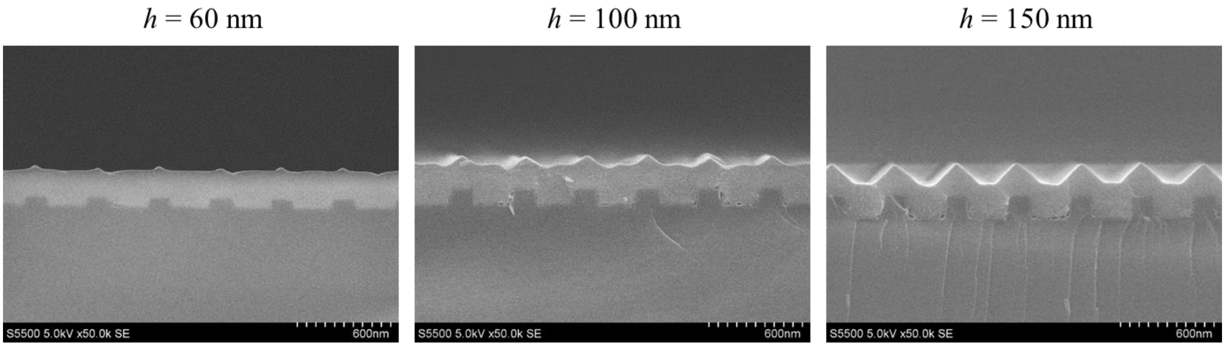

Figure S1 | SEM images of the samples. Cross-sectional SEM images of the LUNAR samples with (p, t) = (400 nm, 250 nm) and h = 60, 100, and 150 nm. The profiles of the surface structures vary according to the nanograting height h.

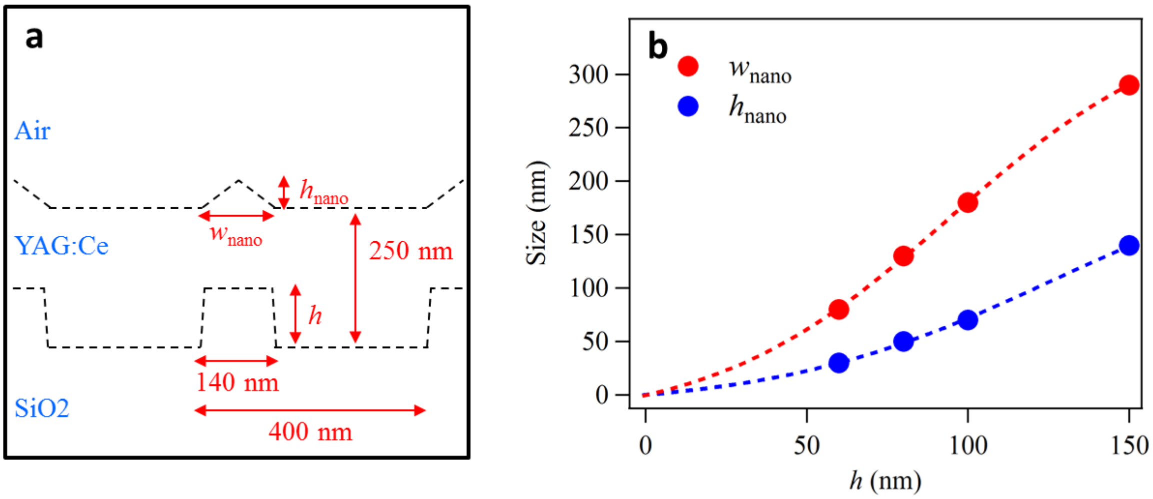

Figure S2 | Calculation model of LUNAR samples. a, Model structure of the LUNAR sample. The model for the nanograting substrate is determined from the SEM image. The surface structure, which varies according to the nanograting height h, is approximated to be triangular in shape, using two design parameters: Base w_{nano} and height h_{nano}. **b,** base w_{nano} and height h_{nano} versus nanograting height h extracted from the SEM images are shown in Fig. S1. The dotted lines are interpolating curves.

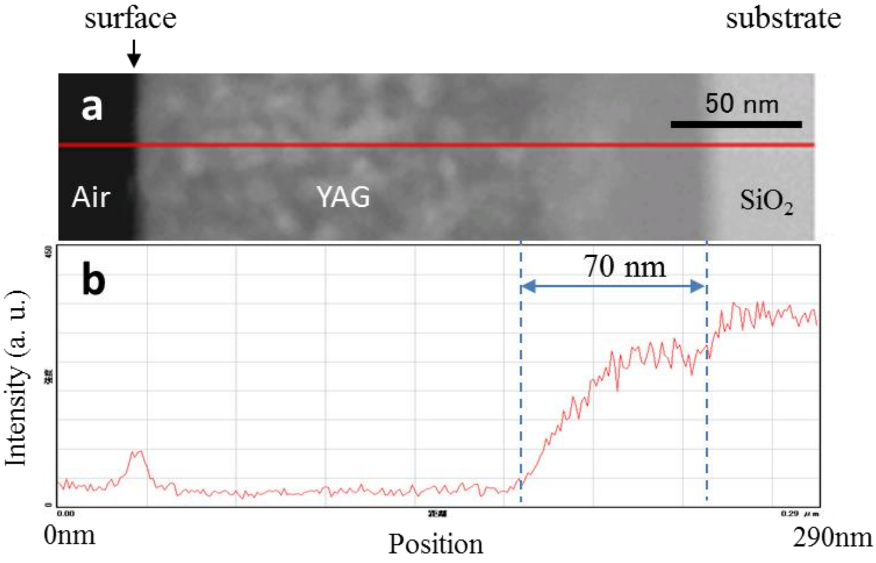

Figure S3 | Material composition of the sample. a, TEM image of YAG:Ce film deposited on flat silica substrate. **b,** The detected signal of Si atoms. Si atoms are diffused from the silica substrate to the YAG:Ce film within ~70 nm from the substrate.

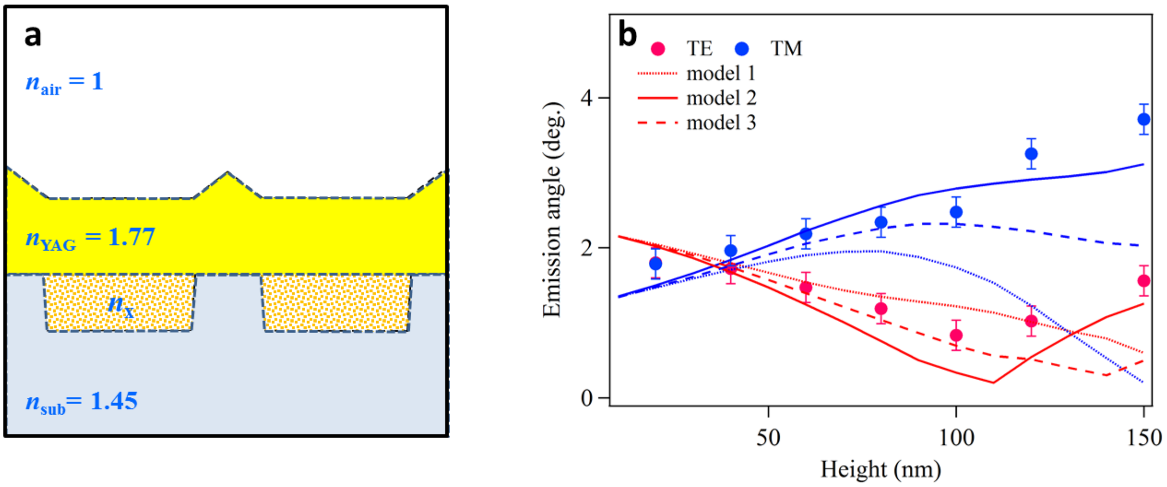

Figure S4 | Refractive indices of the model. a, The refractive indices of the model materials: Air (n_{air} = 1), silica (n_{sub} = 1.45), YAG:Ce (n_{YAG} = 1.77) near the surface and YAG:Ce (n_x) embedded in the nanograting. The value of n_x is different for Models #1 to #3. **b,** The angle of LUNAR emission at a wavelength of 633 nm. Symbols represent the experimental results, and the dotted, solid and dashed lines indicate the calculated result of Models #1, #2 and #3, respectively.

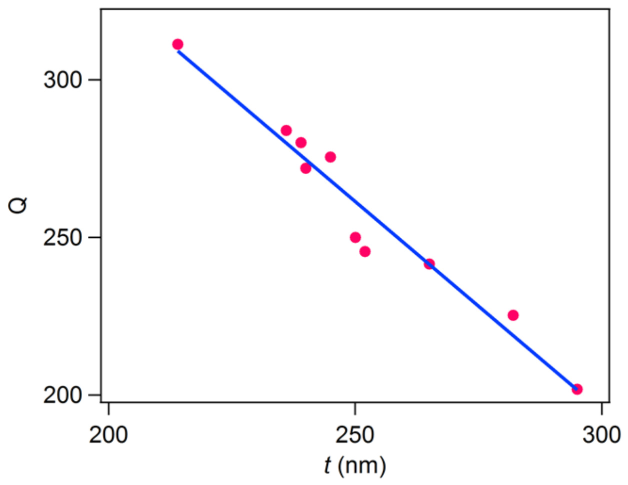

Figure S5 | Dependence of quality factor on YAG:Ce thickness. Quality factor versus YAG:Ce thickness *t* of LUNAR samples. Symbols represent measured quality factors from scattering experiments. The line is added simply to guide the eye.
